# Genome-Wide Tool for Sensitive de novo Identification and Visualisation of Interspersed and Tandem Repeats

**DOI:** 10.1177/11779322241306391

**Published:** 2024-12-18

**Authors:** Ruslan Kalendar, Ulykbek Kairov

**Affiliations:** 1 Helsinki Institute of Life Science (HiLIFE), University of Helsinki, Helsinki, Finland; 2 Laboratory of Bioinformatics and Systems Biology, Center for Life Sciences, National Laboratory Astana, Nazarbayev University, Astana, Kazakhstan

**Keywords:** Direct and inverted repeats, interspersed elements, minisatellite repeats, mobile genetic elements, repeats analysis tool, tandem repeats

## Abstract

Genomic repeats are functionally ubiquitous structural units found in all genomes. Studying these repeats of different origins is essential for understanding the evolution and adaptation of a given organism. These repeating patterns have manifold signatures and structures with varying degrees of homology, making their identification challenging. To address this challenge, we developed a new algorithm and software that can rapidly and accurately detect any repeated sequences de novo with varying degrees of homology in genomic sequences in interspersed or clustered repeats. Numerous forms of repeated sequences and complex patterns can be identified, even for complex sequence variants and implicit or mixed types of repeat blocks. Direct and inverted-repeat elements, perfect and imperfect microsatellite repeats, and any short or long tandem repeat belonging to a wide range of higher-order repeat structures of telomeres or large satellite sequences can be detected. By combining precision and versatility, our tool contributes significantly to elucidating the intricate landscape of genomic repeats.

## Introduction

Repeated sequences are functionally ubiquitous structures found in all genomes (from viruses and prokaryotes to eukaryotes), with higher abundance, especially in noncoding eukaryotic sequences.^1^ Genomic repeats represent integral structural elements across all genomes and contribute to their functional complexity.^2^ As 1% of genomic DNA in the human genome is dedicated to protein-coding sequences, various researchers have suggested that the mobile genetic elements and the other repetitive sequences that constitute the so-called ‘noncoding DNA’ are where the entire evolutionary history of these genomes may be linked.^3-6^ A tandem repeat (TR) is a DNA sequence in which a pattern of 2 or more different nucleotides is repeated one or more times consecutively. The TRs are found throughout the genome and can vary in length from a few base pairs to several kilobases.^7^ Eukaryotic ribosomal RNA (rDNA) gene families typically display TR structures and are present in long arrays. In most eukaryotic genomes, rDNA genes are clustered in long TRs at one or several loci.^8^ Variation in copy number is a common characteristic of rDNAs and has been reported in many organisms. Using rat lines as an example, we previously observed that the copy number of animal genes is also highly variable.^9^ The diversity of TR forms makes identification challenging, as the origin of their development is different. The 2 other forms of TRs are monomeric arrays and higher-order repeats (HORs).^7,10^ Monomeric arrays are formed when TRs occur as individual copies of the same sequence and are repeated one after the other. A TR set is also represented by more complex forms of sequences,^11^ similar to tandemly repeated Alu elements (belonging to an order of nonautonomous retroelements termed short interspersed elements) and 5S rRNA genes. Mobile genetic elements are ubiquitous in eukaryotes and are present in high copy numbers in most genomes, making them significant constituents of eukaryotic genomes.^12,13^ Retrotransposons are substantial elements of interspersed and clustered TRs in all eukaryotic genomes.^14^ One of the remarkable features of retrotransposons is their high copy numbers. This abundance is believed to be due to their ability to replicate and insert into new genomic locations. In addition, the primary order of these elements in various plant species is long terminal repeat (LTR) retrotransposon multiple extended tandem arrays.^15^ Diverse families of inverted-repeat elements, such as the miniature inverted-repeat transposable element (MITEs) family, which belong to a group of nonautonomous class II transposable elements (DNA sequences), are especially typical of the plant genome.^16^ In addition to other mobile elements, MITEs can also form extended TRs that are not detected using next-generation sequencing but can be detected using third-generation sequencing, such as Oxford Nanopore Technologies (ONT) or Pacific Biosciences Sequel systems. The presence of diverse families of inverted and direct repeat elements in genomes is associated with responses to environmental stress and diseases. This may enable genomes to adapt rapidly to new challenges, such as pathogens, climate variations, and other stressors. Detecting repetitive genomic sequences is challenging, and many algorithms are available.^17-23^ These repetitive patterns are characterised by manifold distinct signatures and intricate architectures with varying degrees of homology, making identification difficult.^24^ We developed an advanced tool that detects de novo diverse forms of direct and inverted repeated sequences within genomic compositions. This tool recognises interspersed and clustered repeats, encompassing a broad spectrum of variations, including nested repeat patterns. This enabled the identification of inverted-repeat elements, perfect and imperfect microsatellite repeats, and diverse short and long TRs, thus spanning a comprehensive array of configurations with varying degrees of homology and repeat frameworks of extensive satellite sequences. Our tool offers a robust and rapid solution for comprehensively detecting genomic repeats, even in intricate sequence variations and complex repeat architectures.

## Design and Implementation

We developed an easy-to-use command line and versatile application in Java that can identify various forms of direct and inverted repeat sequences that can be applied to any target genome. Our tool is implemented in Java (requires Java Platform, Standard Edition 23 or higher). User-friendly and accessible genomic repeat analysis software can help researchers identify repeat sequences efficiently and accurately in genomic data, which can further advance the understanding of the role of genomic repeats in biology. The tool runs locally, allowing users to analyse their data (a single or all files in the target folder). It takes the genomic sequence(s) and several parameters as input, defining the length of the k-mers and the type of analysis. In the first stage of the algorithm, identical k-mers that match in different regions of the sequences are lengthened pairwise by the maximum length for them.

In the initial stage of repeat identification, the software uses sequence screening for size-defined k-mers and identifies repeating regions of different lengths in the target sequence. The adjacent repetitive areas are combined to determine the boundaries of all types of repeats. Repeats shorter than the specified filter size will be ignored in further analysis. At the same time, a masked sequence is generated in which regions with repeats are highlighted by lowercase letters. After identifying all repeats and defining their boundaries, the software determines the homology of each repeat to other repeats. The identification of homologous sequences is achieved through the screening of matching k-mers. The minimum similarity of homologous sequences can be at least 60%. Homologous sequences are clustered together and will be output as a separate cluster. The result will be saved in a text file containing a list of clusters and coordinates of repeating blocks in each cluster. In the current version, k-mers should be identical and can be used as ‘seeds’ to detect homologous sequences. The default length of k-mers is 12 nucleotides but can be as short as 5. The recommended minimum size of k-mers is 18 or longer for large genomes. Homologous fragments are searched for both in the same orientation and for a complementary chain.

In the ‘quick search’ mode, the identified homologous sequences are subsequently grouped into their respective clusters. Although this mode is the fastest, there will potentially be some false clustering of these repeats for large genomes with different types of mixed repeats. This mode is recommended by default for analysing large genomic sequences (eg, chromosomes) to obtain an overview of global genomic rearrangements.

Under profile search mode (quick = false), the homologous sequences thus detected are grouped into an individual cluster and will not be clustered with overlapping sequences from different clusters. This mode allows the study of the complex structure of genomic repeats, including large telomeric or chromosomal duplications and rearrangements. This mode is relatively fast, does not use inter-clustering, and is the safest to analyse genomic rearrangements and general analysis of repetitive elements in the genome. Using this mode, it is possible to observe which clusters overlap and are related.

This mode enables the separation of different types of repeat sequences, a critical feature of the analysis since sequences typically consist of multiple repeat types. The software clusters repeated blocks for smaller genomes, such as those of bacteria, fungi, protozoa, and other target sequences. Overlapping blocks are automatically considered homologous and grouped into a common cluster. At this stage, however, different repeat elements may become mixed. This misclustering is especially common in eukaryotic genomes, where mixed elements can be distinguished by their copy number, as mixed repeats are less likely to be repeated elsewhere.

Many mobile elements contain low-complexity sequences, such as microsatellites or poly-A-rich regions, which must be accurately linked to their corresponding genetic elements and distinguished from other genome sequences. The algorithm effectively separates scattered low-complexity sequences from those associated with specific genetic elements. Furthermore, analysing the sequence complexity allows for the identification of perfect and imperfect microsatellite repeats, as well as short or long TRs.

The result is saved in a GFF (9-column, table-delimited) text file, a sequential list of clusters of homologous sequences indicating the start and end of each site, its orientation, and the sequence itself. When visualising the results, sequences in the forward orientation are indicated by blue, and those in the opposite orientation are indicated by red (mode with quick = false) in the output graphic PNG file.

The main achievement of our software is a rapid analysis of any repetitive sequences in both short and large chromosomes. The obtained masked sequence, GFF, and graphical PNG files allow for an understanding of the overall structure of the target sequence for any repeats and the evolution of these repeats. It is possible to trace different types of repeats and their relationship at the genomic level. In a graphical PNG file, each cluster is localised in a separate line along the current sequence. In the sequence scale, the first cluster appears at the top, with subsequent clusters arranged beneath it. If a cluster contains homologous sequences in different orientations, they are colour-coded: blue for forward orientation and red for inverted orientation. In cases where inverted repeats are analysed (with quick = false), both forward and reverse orientations are displayed but are grouped into separate clusters. This feature allows the detection of homologous sequences located in different parts of the sequence.

Chromosome analyses performed with the profile mode (quick = false) enable a detailed distinction of the repeat profile at the genomic level, revealing its characteristic repetitive structure. Notably, this level of repeat profiling is a unique capability of the software, unmatched by any other existing tools.

In addition, our algorithm eliminates the need for extensive alignment matrix calculations and does not rely on prior knowledge of repeat patterns. By employing a simple and efficient approach based solely on k-mer screening, it maps sequences and determines sequence similarity with high precision. The algorithm swiftly defines the boundaries of all repeats and calculates homologous distances between them using k-mer distance. Importantly, there is no size limitation on the repeats that can be detected, and the algorithm accounts for nucleotide substitutions between repeated copies.

Unlike traditional methods, our algorithm accurately identifies repeated sequences without relying on frequency pattern analysis, alignment, or suffix tree construction. It is designed to autonomously detect repeated sequences, regardless of their divergence or length, making it highly versatile. This adaptability allows the software to excel in detecting a wide range of repeat structures, including mobile elements, ribosomal genes, large genomic rearrangements, telomeric and centromeric regions, and other direct and inverted repeats. These repeated sequences may be situated at varying distances from each other and oriented in different directions, yet the algorithm consistently identifies them with precision.

The masked sequence contains all variants of repeat sequences without any initial classification. The identified related repeat sequences can serve as a platform for subsequent classification, allowing researchers to determine their relationships to established repeat element groups or even discover novel repeat types. To streamline this process, the software immediately categorises all identified repeats into clusters based on shared sequence features. However, it does not perform classification or detailed analysis of de novo repeats associated with genes, gene families, or mobile elements.

Classifying repeats into specific families remains one of the most challenging aspects of repeat annotation, as repeats within a genome can often be misgrouped, especially in the case of hybrid elements. This misclassification is common in eukaryotic genomes. Therefore, a more specialised analysis of each repeat type must be conducted using external tools. For example, established tools like RepeatMasker^25^ or CENSOR tool^26^ can be employed to compare query sequences against reference repeat collections (eg, https://www.girinst.org/).

While the current software does not handle detailed repeat family classification, the data generated from the clustered repeats can be used for further in-depth analysis. In profiling mode (quick = false), the software excels at analysing complex ‘repeat within repeat’ patterns and intricate sequence variants, including implicit and mixed repeat blocks. This advanced capability is particularly useful for detecting highly complex sequence variations and mixed repeat types.

Furthermore, the software provides accurate classification of perfect and imperfect microsatellite repeats, as well as short and long TRs that belong to various HOR structures, such as telomeres or large satellite sequences. This multifaceted functionality makes the software a comprehensive tool for researchers investigating the complex landscape of genomic repeats.

## Results

### Unveiling complex TRs in genomes: insights from our analysis

**Figure 1.**
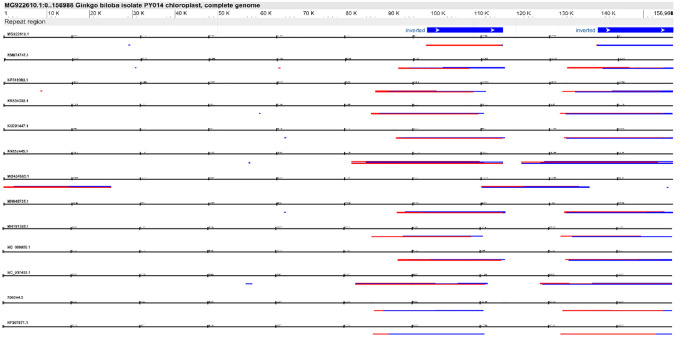
Genome-wide repeat profiling of chloroplast genomes was performed to identify interspersed and clustered repeats of (*Tectona grandis* (HF567871.1), *Phleum alpinum* (KM974747.1), *Diplopanax stachyanthus* (KP318983.1), *Tanaecium tetragonolobum* (KR534325.1), *Zea nicaraguensis* (KU291447.1), *Samanea saman* (KX852445.1), *Artocarpus heterophyllus* (MG434693.1), *Ginkgo biloba* (MG922610.1), *Panax ginseng* (MH049735.1), *Ribes fasciculatum* (MH191388.1), *Lolium perenne* (NC_009950.1), *Adenocalymma acutissimum* (NC_037455.1), and *Nicotiana tabacum* (Z00044.2)). The upper part of the figure contains a graphical analysis of this site obtained from NCBI GenBank (*Ginkgo biloba*, MG922610.1) with a graphical view (https://www.ncbi.nlm.nih.gov/nuccore/MG922610.1?report=graph). The lower part of the figure was obtained from our study to identify interspersed and clustered repeats (analysis parameters for profile searching: k-mer = 19; minimum repeat block = 90).

To illustrate the functionality of our tool, we performed analyses on different genomic sequences from different taxonomic groups of eukaryotes (animals, birds, plants, insects, fungi, and protists), bacteria, plastids, and giant viruses. We compared the de novo identification of repeat sequence with REPuter (https://bibiserv.techfak.uni-bielefeld.de/reputer),^27^ RED,^21^ RECON, and RepeatScout^28,29^ (Table 1). For comparison, the ability of RepeatMasker to identify only already characterised repeats was considered. For example, we showed that 2 inverted repeats in the chloroplast genome have a more complex structure, corresponding to inverted repeats within direct repeats (Figure 1). REPuter, a widely used software, employs a suffix tree algorithm to identify approximate repeats within genomic sequences. It has been particularly effective in detecting TRs and palindromic sequences. RED (REpetitive DNA detector) uses a graph-based approach to identify repeats, focusing on exact and approximate matches. RECON (REpetitive DNA CONsensus) employs a consensus sequence approach to identify repeats, aiming to provide a comprehensive view of repetitive elements within genomic sequences. Generic Repeat Finder (GRF) is a software for identifying repetitive elements within genomic sequences. It employs a simple yet efficient algorithm that scans the input sequences for repetitive patterns and reports their occurrences. However, GRF may lack the sophistication to handle complex repeat structures and may not provide comprehensive insights into the organisation and distribution of repetitive elements within genomes. RepeatScout, on the contrary, is a more advanced software tool designed to identify repeats through de novo discovery. It employs a suffix tree-based algorithm to identify significant repeats within genomic sequences without relying on pre-existing repeat libraries. RepeatScout excels in detecting novel repeat families and offers valuable insights into the evolutionary dynamics of repetitive elements within genomes. Furthermore, we considered the performance of RepeatMasker, a widely used tool for identifying repeats based on a library of known repeat elements. We evaluated its ability to accurately identify previously characterised repeats within the genomic sequences under analysis. Through our comparative analysis, we aimed to provide insights into the strengths and limitations of each software solution, with a focus on their applicability across different taxonomic groups and the comprehensiveness of repetitive element identification. Compared with existing software, our algorithm does not use multiple alignments for the de novo identification of repeat sequences; the algorithm eliminates the need for extensive alignment matrix calculations and suffix tree construction. The size of repeats that can be detected is unlimited, and the algorithm accounts for nucleotide substitutions between repeated copies. It is designed to autonomously detect repeated sequences regardless of variance in divergence and length. At the same time, it is possible to identify all repetitive sequences with a copy number of at least 2 copies and a homology of at least 60%. Although the algorithm concept for identification of repeat sequences may be similar to existing software, implementing these algorithms is different. The main advantage of our approach is the implementation of a graphical representation of identified repeats at the genomic level. Repeats can have an internal structure consisting of other repeats in both forward (highlighted in blue) and reverse orientation (highlighted in red). If we trace the structure of genomic repeats at the chromosomal level, we can determine their relatedness and evolutionary divergence without annotating their classification. Genomic blocks formed by duplication and located in different parts of chromosomes and orientations accumulate multiple changes in the process of evolution. However, we can identify the relatedness of these genomic blocks by using certain structural patterns. Our algorithm identifies low-complexity repetitive sequences characteristic of perfect and imperfect microsatellite repeats and any short or long TRs belonging to a wide range of repeat structures organised from telomeres or large satellite sequences and extended genomic repeats, which can make up the majority of chromosomal sequences. Therefore, in this tool, we do not limit ourselves to a particular repeat type but identify all available repetitive sequences, regardless of whether they belong to one repeat type. The examples below demonstrate genomic analyses for different organisms and structural sequences. Table 1 compares the software available for de novo identification of repeat sequence analysis. Our tool does not require additional steps, such as the individual compilation of certain blocks for a particular operating system or the installation of additional libraries, as it is a standard application for the Java Platform. The direct launch of the application is made in the command line with the call of the application and the target file or the path to the directory with files: java -jar Repeater2.jar <inputfile>.

**Table 1.** The programme’s main features include a comparative analysis of genome-wide tools for de novo identification of interspersed repeats.

Evaluation criteria	Repeater2	REPuter	RED	RECON/RepeatScout
Environmental	Java	Web, Linux, OSX, Solaris, Irix, and Alpha	C++, OS: Mac, Unix	C, Perl scripts
Genome limitation	No limitation, but it depends on memory capacity	5 Mb and a maximum of 5000 repeats	no limitation, but it depends on memory capacity	10 Mb
K-mer length	5 to any (default 12)	10	12-15	30
Pairwise local alignments	Alignment-free	Yes	Yes	Yes
Short, long, and very long tandem repeats	Yes	No	No	No
Microsatellites (2-6 bp) and telomeres (5-10 bp)	Yes, perfect and imperfect	No	Yes	No
Mobile elements	Yes	No	Yes	Yes
Inverted and palindromic repeats	Yes	Yes	Yes	No
Gene and chromosomal duplications	Yes	Yes	No	No
‘Repeat within repeat’	Yes	No	No	No
String homology	>60%	>67%	unknown	unknown
Repeat masking	Yes	No	Yes	No
Classification	Yes	No	No	No
Machine learning	No	No	Yes	No
Visualisation repeat profile	Yes	No	No	No
Relative calculation speed	Quick	Slow	Slow	Slow
Level of execution	Any user	C compilation, programmer required		

This is a significant advantage over existing applications that require skill and are challenging for a user to deploy. The main differences concern the algorithms used in this software and the potential for further development. Our tool also leaves the potential for further development and improvements in the annotation of repetitions and their classification. Masked sequences can be used for subsequent analysis of homologous repeats within a particular chromosome and between different chromosomes of the same or other related species, followed by their annotation. Graphical representation of individual chromosomes for whole genome analysis will be a practical comparative and evolutionary analysis of a particular species and related species. Thus, mobile elements are identified in genomic sequences by their characteristic structural elements, namely LTRs, primer binding sites (PBS), and specific genes (gag, rt, int).^30^ However, these limit the search for new types of mobile repeats and related elements. Therefore, the primary analysis should be based on identifying all repetitive sequences and analysing these repeats and their annotation.

Another example of complex direct repeats is the LTRs in endogenous retroviruses and retrotransposons, which also have a loop structure characteristic of inverted repeats despite being direct repeats.^15^ This is a remarkable phenomenon, probably characteristic of most LTR-retrotransposons, in which long direct repeats are both inverted repeats, forming a loop structure between distal repeats. We observed loop structure patterns for direct repeats exemplified by MITEs in plant genomes and genomic repeats in eukaryotes and prokaryotes. Thus, most tandem elements are complex mixed repeated sequences with a ‘tandem within a tandem’ pattern.

**Figure 2.**
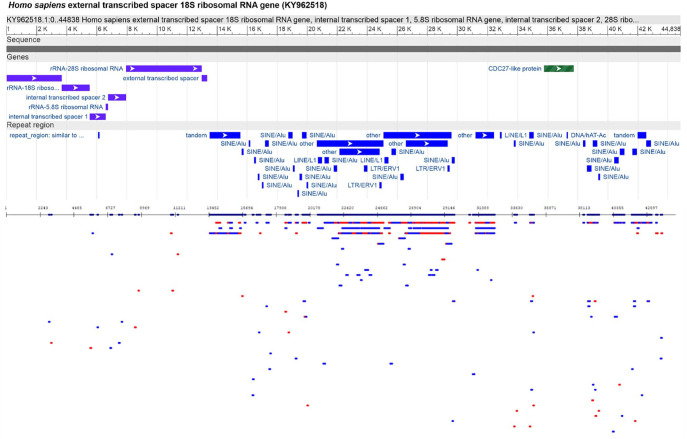
Comparative analysis of a region (KY962518) of the *Homo sapiens* genome containing the external transcribed spacer 18S ribosomal RNA gene, internal transcribed spacer 1, 5.8S ribosomal RNA gene, internal transcribed spacer 2, 28S ribosomal RNA gene, external transcribed spacer, and CDC27-like protein pseudogene (KY962518). The upper part of the figure contains a graphical analysis of this site obtained from NCBI GenBank with a graphical view (https://www.ncbi.nlm.nih.gov/nuccore/KY962518.1?report=graph). The lower part of the figure was obtained from our study to identify the interspersed and clustered repeats. The fragments identified by RepeatMasker (http://repeatmasker.org) were consistent with our analysis and clustering of similar repeats. Plots containing genes (exons) do not contain repeats or mobile elements. Profile analysis parameters (k-mer = 9; initial length = 50; minimum repeat block length = 100).

This software identifies long tandems, typically either for retrotransposons or ribosomal genes; repeats of any length can be detected locally and throughout the genome. Repeats associated with the chromosome’s centromeric or other structural regions consist of a complex mixture of sequences included in each other or the neighbourhood. For example, chromosome 1A from the fungal genome of *Puccinia triticina* contains very long inverted structures putatively in 2 centromeric regions, which in their structure and length we have not identified in other organisms (Supplemental Figure S1). The cluster of ribosomal genes in the human genome also consists of a cluster of mobile elements unique among animal genomes (Figure 2).

**Figure 3.**
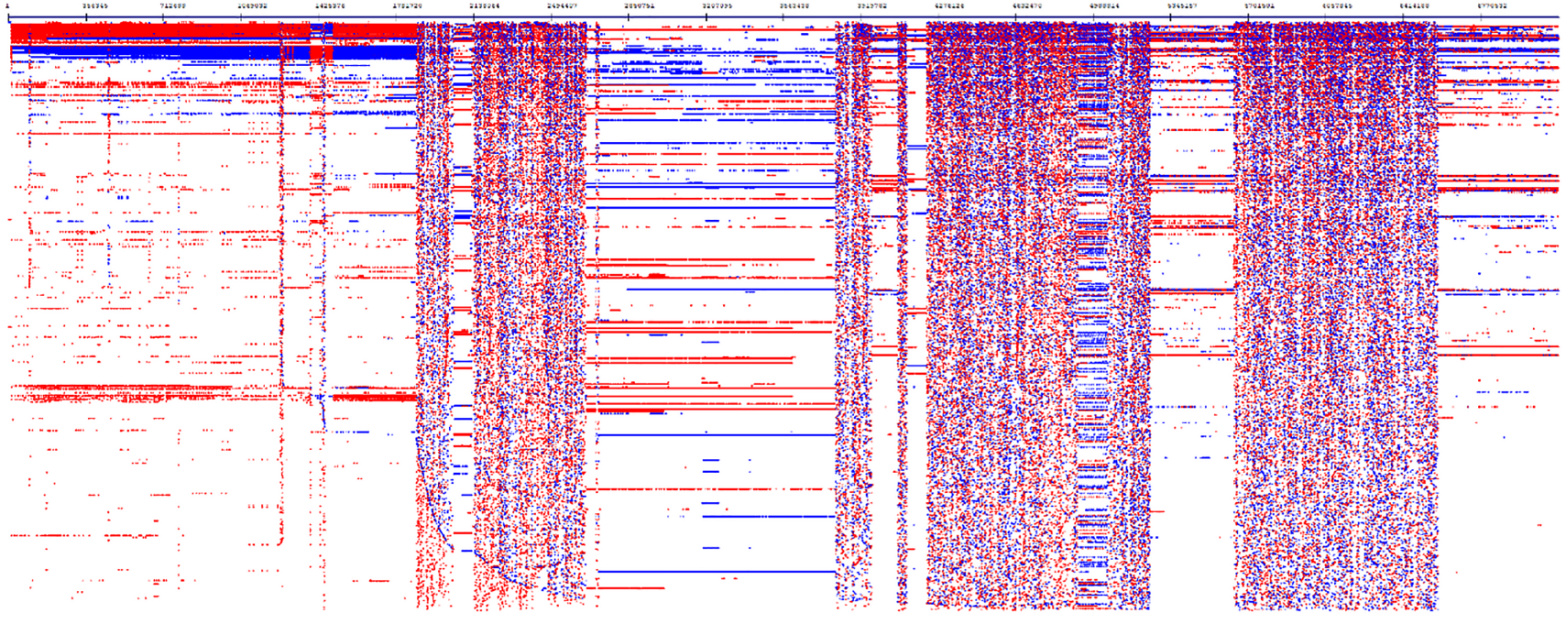
Genome-wide profile of all repeats for the small hive beetle (*Aethina tumida*) chromosome 8 (NC_065442.1), without clustering. The horizontal axis shows the chromosomal sequence, and the vertical axis shows repeats clustered in a single line (profiling analysis parameters: k-mer = 9; initial length = 50; minimum repeat length = 100). Repeat coverage for the chromosome 8 genome was 95.82%. The chromosome 8 genome consists of very long repeats (several million base pairs) that are further duplicated in different parts of the chromosome and undergo divergence, compression, or expansion.

**Figure 4.**
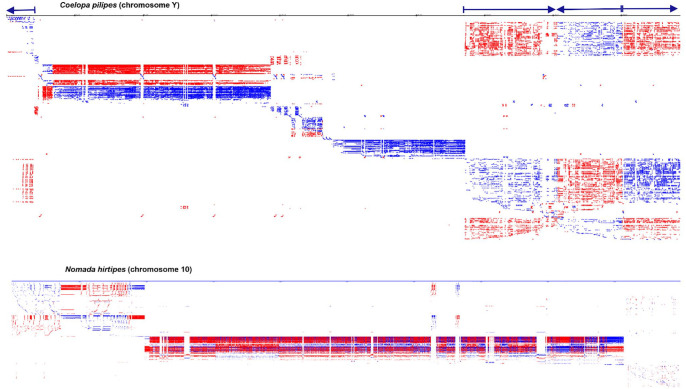
Genome-wide profile of all repeats for insect genomes using *Coelopa pilipes* (chromosome Y, OX376702) and *Nomada hirtipes* (chromosome 10, OX637925) (analysis parameters: k-mer = 12; initial length = 50; minimum sequence length = 150). The high density of long repeats distributed throughout the genomes of these insects suggests a complex parallel evolution of the genomes of these species and, in general, of all insects. Repeat coverage for the *C pilipes* chromosome Y genome was 98.9%; the corresponding value for *N hirtipes* chromosome 10 was 61.02%. For chromosome Y (*C pilipes*), the arrows indicate related blocks that have formed due to duplications. These blocks are arranged in the chromosome in a forward and backward direction.

There is a direct correlation between genome size and the density of repeat forms. Plant genomes contain many repeats, similar to animal and human genomes. A unique feature of plant genomes is the occurrence of TRs of mobile elements, like ribosomal gene clusters. In addition, the main elements of the plant genome are LTR retrotransposons, which are numerous elongated tandem arrays whose copy numbers differ among plant lines or cultivars and are challenging to analyse and sequence. Plant genomes are also characterised by repeats originating from LTR-retrotransposons that form extended tandem blocks.^15^ For insect genomes, telomeric regions are also characterised by the same extended tandem blocks of repeating LTR-retrotransposon identical copies. However, the uniqueness of insect genomes is not limited to the specificity of telomeric regions. Recent studies have examined 601 insect species and found significant differences in the dynamics of mobile genetic elements.^31^ Our software analysed genome-wide de novo identification and visualisation of interspersed and TRs for insect genomes. In the insect genomes, we observed large arrays of diverse repeats of various origins, including very long repeats. The insect genomes of *Coelopa pilipes* (chromosome Y, OX376702) and *Nomada hirtipes* (chromosome 10, OX637925) showed large chromosomal blocks duplicated in different parts of the chromosome. For example, chromosome Y (3 407 776 bp) for *C pilipes* genome consists of repeats on 99.53% (k-mer = 12, Initial string length filter = 50, String length filter = 100, Quick = true). These blocks are complex repeats, some exceeding >100 000 bases in length. Genomic analysis of chromosome 8 of the small hive beetle (*Aethina tumida*) revealed massive duplications, mainly in a directed tandem orientation. Repetitive sequences accounted for at least 60% of the insect genome (Figures 3 and 4). For *C pilipes* (chromosome Y) (Figure 4), the arrows indicate related blocks that formed due to duplications. These blocks are arranged in the chromosome in a forward and backward direction. After duplication, these blocks began to diverge to create a unique pattern. In both chromosomes of these insects, a similar situation was observed for other parts of the chromosome but on a smaller scale, or the rearrangements were multiple and extended, as observed in the central part of chromosome Y (*C pilipes*) and most of chromosome 10 (*N hirtipes*).

**Figure 5.**
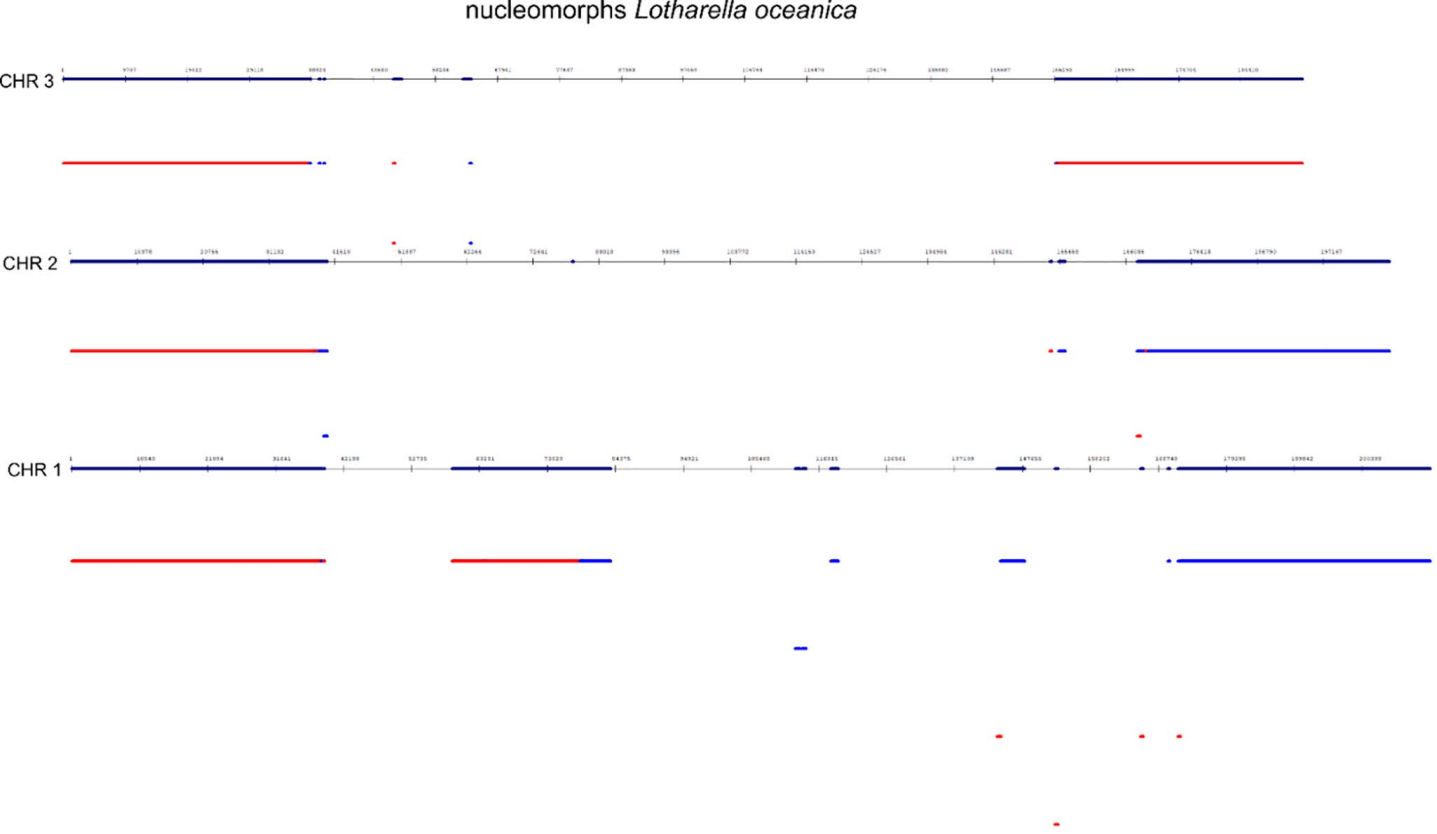
Genome-wide profile of all repeats in the 3 chromosomes of the nucleomorph *Lotharella oceanica* (CP006628). An inverted sequence represented the terminal and concurrent telomeric regions of the nucleomorph. These regions contain identical ribosomal gene sequences, including multiple gene duplications.

Genome-wide de novo identification and visualisation of interspersed and TRs was performed for nucleomorphs. Nucleomorphs represent some of the smallest genomes that have been sequenced and are characteristic of both cryptomonads and chlorarachniophytes, converted to a similar size from larger genomes. The terminal and concurrent telomeric regions of all 3 chromosomes of the nucleomorph *Lotharella oceanica* (CP006628) were represented by an inverted sequence. These regions contain identical sequences for ribosomal genes (eg, for chromosome 2, sequentially from the left part: 28S rRNA [626. . .4069] - 5S rRNA [4497. . .6403] - 18S rRNA [6754. . .7059] and the right side in the opposite orientation: 18S rRNA [201141. . .203047] - 5S rRNA [203165. . .203255] - 28S rRNA [203475. . .206918]). However, the sequence of the ribosomal cluster does not exceed 5000 bases, whereas the inverted end repeat is more than 38 000 bases each (38 447 and 38 023 bases, respectively), including multiple genes. For chromosome 2, with a length of 207 kb, end repeats accounted for 37.4% of chromosome 2. In addition, chromosome 1 had a third large repeat, consisting of end-site genes (Figure 5).

**Figure 6.**
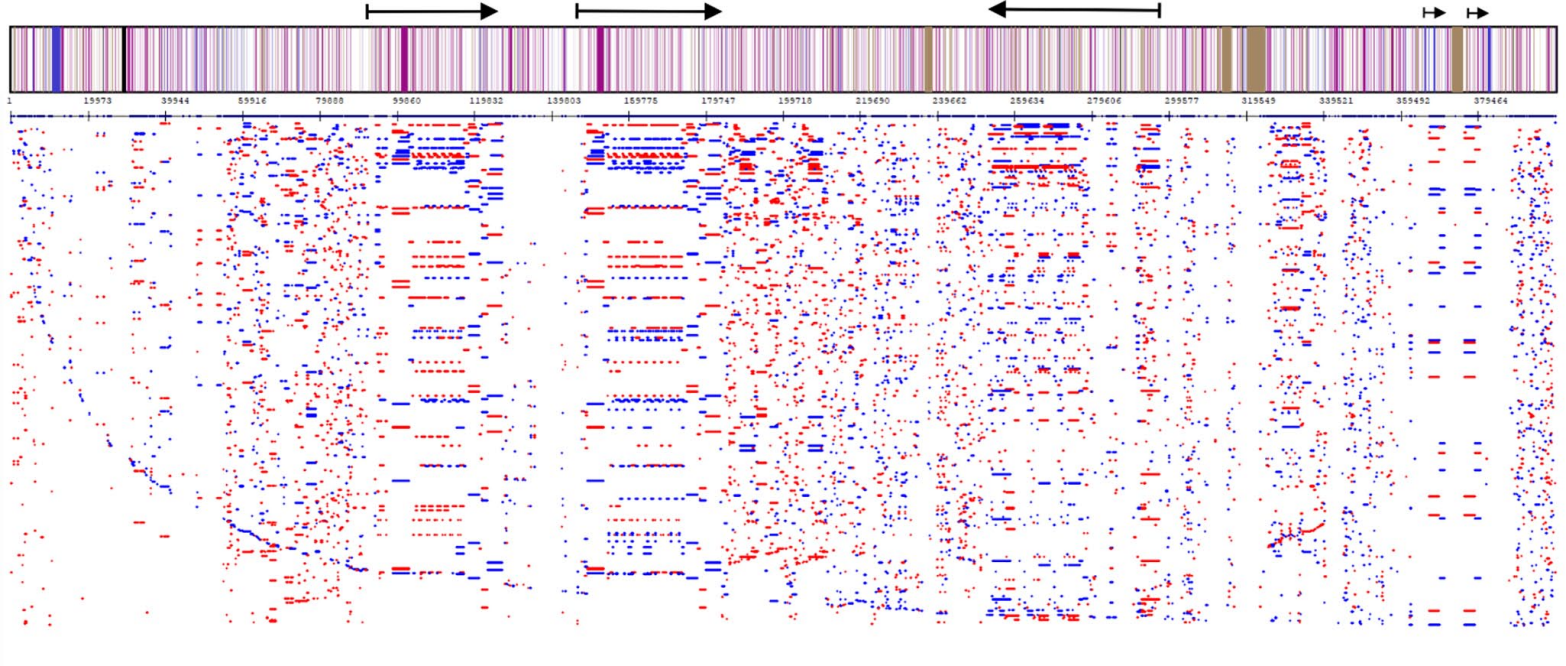
The extrachromosomal replicon of *Amaranthus palmeri* circular DNA (399 435 bp, GenBank: MT025716.1) was analysed by comparison using the CENSOR (https://www.girinst.org/censor/) programme (upper panel). Profile analysis parameters (k-mer = 9; initial length = 50; minimum repeat block length = 100). The eccDNA replicon sequence consists of a complex arrangement of direct and indirect repeat motifs of variable lengths dispersed among retroelements in addition to DNA transposons interspersed by predicted *MITE* and *Helitron* elements. The total length of the repeats was approximately 67% of the whole sequence. Two direct and one inverted block with a length of more than 30 000 nucleotides (2 direct blocks between 99 000 and 129 000 nucleotides and the second 150 000 and 180 000 nucleotides) are well distinguished.

Comparative analysis of the genomes of 8 sequenced *Escherichia coli* strains revealed that the genome contains gene duplications, mobile element variations, remnants of phages, and various regions of unusual composition.^32^ The proportion (%) of repeats among the *E coli* strains compared was of different lengths (Table 2). Our analysis revealed that the total repeat fraction was slightly above 9.4% for the minimal genome of *E coli* strain K-12 substr. MG1655 (NC_000913) (size 4.59 Mb). In contrast, the total repeat fraction was slightly above 20.4% for the most extended genome of strain 97-3250 (NZ_CP027599) (size 5.88 Mb). Thus, variation in the *E coli* genome depends directly on repetitive elements and suggests genome plasticity through processes related to repetitive elements associated with horizontal transfer (Figure 6). Moreover, these repeats are related and have a similar pattern, indicating the related nature of these sequences (likely IS elements). The differences mainly concern the further development of these repeats in the form of accumulation of internal changes and additional insertions, which leads to their expansion and degradation (Figure 6). Intraspecies variation in bacterial genomes can be expressed within quite significant limits. This is similar to what we observed in the genomes of 8 sequenced *E coli* strains, where more than 1 million nucleotides represent repeats of different origins.

**Table 2.** Comparative analysis of the genomes of 8 sequenced *Escherichia coli* strains (repeats searching parameters: k-mer = 9, initial sequence length = 50, minimal sequence length = 150).

Strain and GenBank accession ID	Genome length, bp	Proportion (%) of total repeats
97-3250 (NZ_CP027599)	5 942 969	20.47
EH2252 (NZ_AP027176)	5 770266	19.57
O157:H7 Sakai (NC_002695)	4 641 652	15.72
NCTC9112 (NZ_LR134079)	5 468 700	14.19
O103:H2 12009 (NC_013353)	5 449 314	16.41
O103:H2 FWSEC0007 (NZ_CP031908)	5 397 605	15.96
14EC020 (NZ_CP024138)	4 914 884	10.11
K-12 MG1655 (NC_000913)	4 641 652	9.47

**Figure 7.**
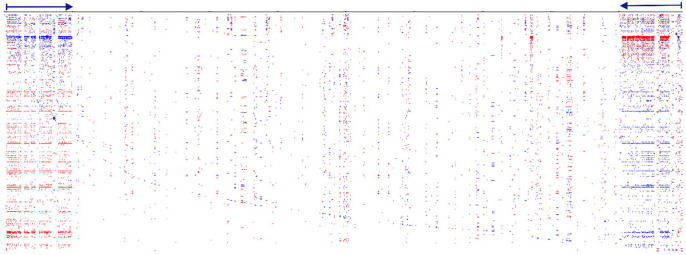
Genome-wide profile of all repeats for the complete genome of the *Bodo saltans* virus (MF782455) family Mimiviridae giant viruses with clustering of common sequences. The horizontal axis shows the chromosomal sequence, and the vertical axis shows the repeats clustered in a single line. The arrows show the extended inverted repeat at the ends of the virus genome, and each of these repeats consists of smaller repeats. The further evolution of these repeats was different, and the integrity of the repeats began to undergo divergence in the form of multiple rearrangements and insertions of other sequences that did not belong to a given repeat. Profile analysis parameters (k-mer = 9; initial length = 50; minimum sequence length = 100; quick = false).

The equally complex structure of the giant virus (*Bodo saltans* virus) genome of the Mimiviridae family contains numerous repeats of genes and other elements, including telomeric repeats, similar to eukaryotic chromosomes.^33^ The *B saltans* virus genome is characterised by the telomeric region’s large, inverted repeat that has undergone divergence. The further evolution of these repeats was different, and the integrity of the repeats began to undergo divergence in the form of multiple rearrangements and insertions of other sequences that did not belong to a given repeat (Figure 7).

Using CENSOR software, we analysed the repeats for the *Amaranthus palmeri* extrachromosomal circular DNA (eccDNA) replicon (Supplemental Figure S2).^34^ The eccDNA replicon sequence consists of a complex arrangement of direct and indirect repeat motifs of variable lengths dispersed among retroelements and DNA transposons interspersed by predicted *MITE* and *Helitron* elements.

The task of clustered regularly interspaced short palindromic repeat (CRISPRs) array detection is to search for short, direct repeats (approximately 20-50 bp) in archaeal and bacterial genomes that can form CRISPR array repeats and then evaluate the identified putative array.^35^ Identification criteria included the length of the repetitive sequence and the similarity between repeats within the array. Therefore, we used parameters for short fragments not exceeding 20 to 30 nucleotides to identify the entire repeat-spacer array of the CRISPR loci. Supplemental Figure S3 shows an example of identifying the CRISPR repeat-spacer array in the *E coli* genome, where the homology between repeats ranged from 87% to 100%. Additional examples are provided in the Supplementary Material.

## Conclusion

Despite the exponential growth in the diversity of genomes, it is evident that the rate at which the scientific community contributes to repeated element databases has not kept up, hindering efficient annotation and comprehensive study of repeated elements across various groups. Thus, there exists a significant opportunity for the field of biodiversity genomics to thoroughly embrace and prioritise research on repeated elements. We developed a unique and flexible tool for the de novo identification of diverse forms of direct and inverted repeated sequences interspersed and clustered in genomic sequences. The algorithm can identify all types of repeated sequences, including perfect and imperfect microsatellite repeats and any short TR belonging to a wide range, organised into HOR structures of large satellite sequences and telomeres. This software is a susceptible and automated method for repeated sequence identification. Classification and comprehensive analysis of the detection of de novo repeats associated with genes and their families and mobile elements were not performed using our tool. The main features of our software include a novel, rapid approach that does not rely on alignment or suffix tree construction, versatility in identifying various types of repeats, ability to handle large genomes, and visual representation of repeat profiles. Our software was tested on animals, insects, plant chromosomes, chloroplasts, and prokaryotic and giant virus genome sequences. It was rapid, efficient, and simple to use, with a user-friendly interface. Novel complex repeats were predicted in multiple genomes. In addition, the software can provide information on the location and distribution of these repeat sequences within the genome. This can help researchers identify potential regulatory regions or areas prone to genetic instability. By providing a user-friendly interface and comprehensive analysis options, this accessible software greatly facilitates the study of genomic repeats and their impacts on biological processes. Furthermore, the ability to run the tool locally ensures data privacy and allows customised analysis parameters tailored to specific research needs.

## Supplemental Material

sj-docx-1-bbi-10.1177_11779322241306391 – Supplemental material for Genome-Wide Tool for Sensitive de novo Identification and Visualisation of Interspersed and Tandem Repeats Supplemental material, sj-docx-1-bbi-10.1177_11779322241306391 for Genome-Wide Tool for Sensitive de novo Identification and Visualisation of Interspersed and Tandem Repeats by Ruslan Kalendar and Ulykbek Kairov in Bioinformatics and Biology Insights
