# Supplementary material for: Genome-Wide Tool for Sensitive de novo Identification and Visualisation of Interspersed and Tandem Repeats
Source: Bioinform Biol Insights. 2024 Dec 18;18:11779322241306391. doi: 10.1177/11779322241306391 (PMC11656428; doi:10.1177/11779322241306391)
Supplement: sj-docx-1-bbi-10.1177_11779322241306391 – Supplemental material for Genome-Wide Tool for Sensitive de novo Identification and Visualisation of Interspersed and Tandem Repeats [file sj-docx-1-bbi-10.1177_11779322241306391.docx]

**Supplemental Material**

**
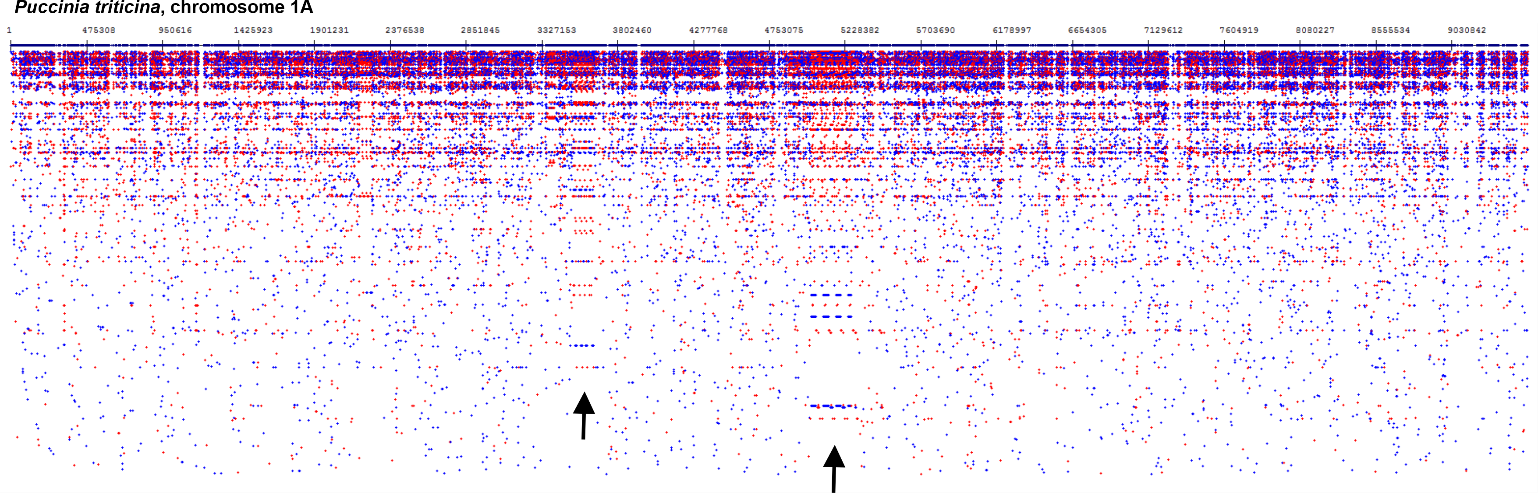
**

**Figure S1**. The fungal chromosome 1A for the genome of *Puccinia triticina* (CM033283) contains very long inverted structures putatively in two centromeric regions (indicated by arrows), which are unusual in their structure and length.


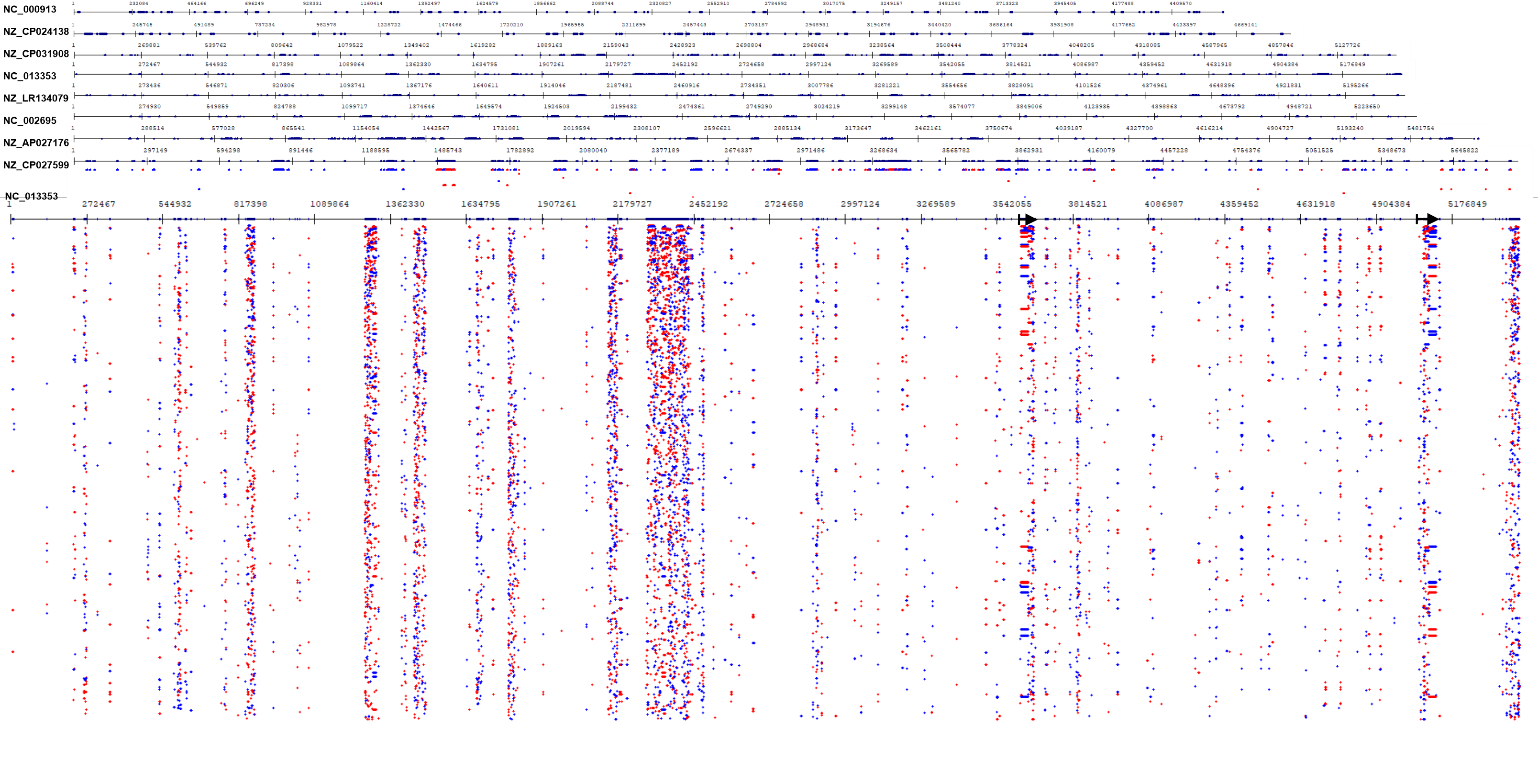


**Figure S2**. Comparative analysis of six sequenced *Escherichia coli* genomes (NC_000913, NZ_CP024138, NZ_CP031908, NC_013353, NZ_LR134079, NC_002695, NZ_AP027176, and NZ_CP027599). This analysis revealed that the genome contains numerous and extensive gene duplications, mobile element variations, phage residues, and several regions of unusual composition. The genome is shown as a horizontal line and regions with repeats are highlighted by blue blocks on this line. Genome sizes were adjusted according to their length. The shortest genome of strain K-12 MG1655 (NC_000913) was 4.59 Mb and the longest genome of strain 97-3250 (NZ_CP027599) was 5.88 Mb. Analysis parameters (kmer=9; Initial Length=50; Minimum Sequence Length=150; QuickSearch=False).


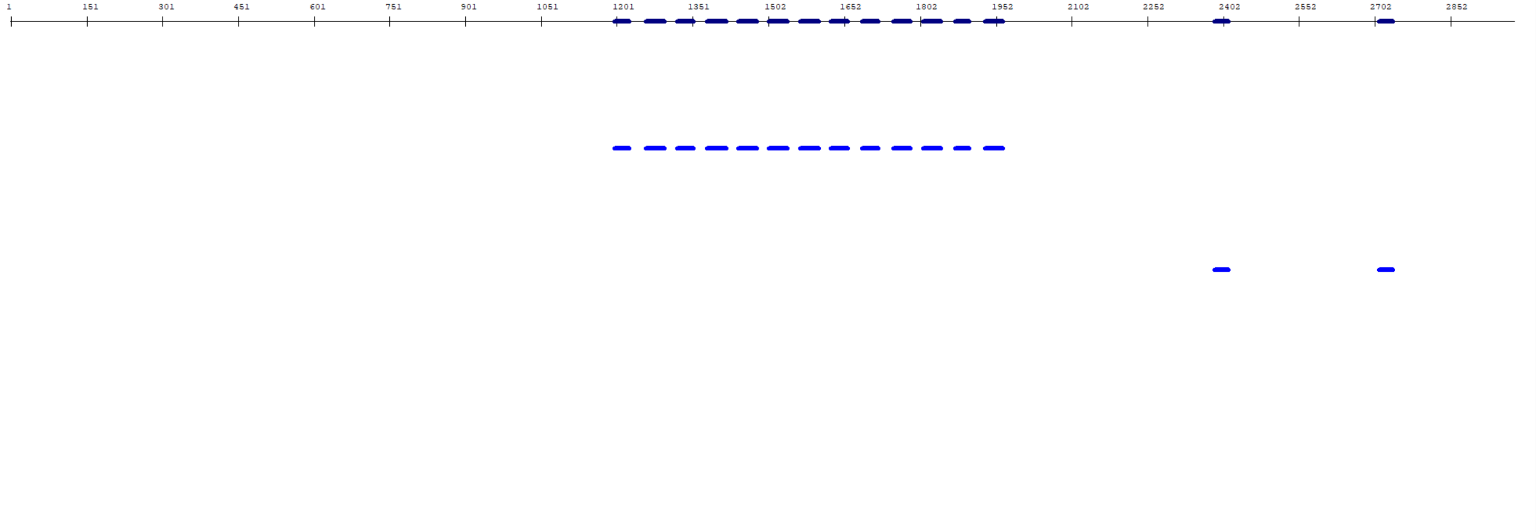


**Figure S3**. Identification of CRISPR-associated endoribonuclease repeat arrays in the *Escherichia coli* genome (CP128218). Analysis parameters (kmer=9; Initial Length=30; Minimal Sequence Length=30; QuickSearch=False).


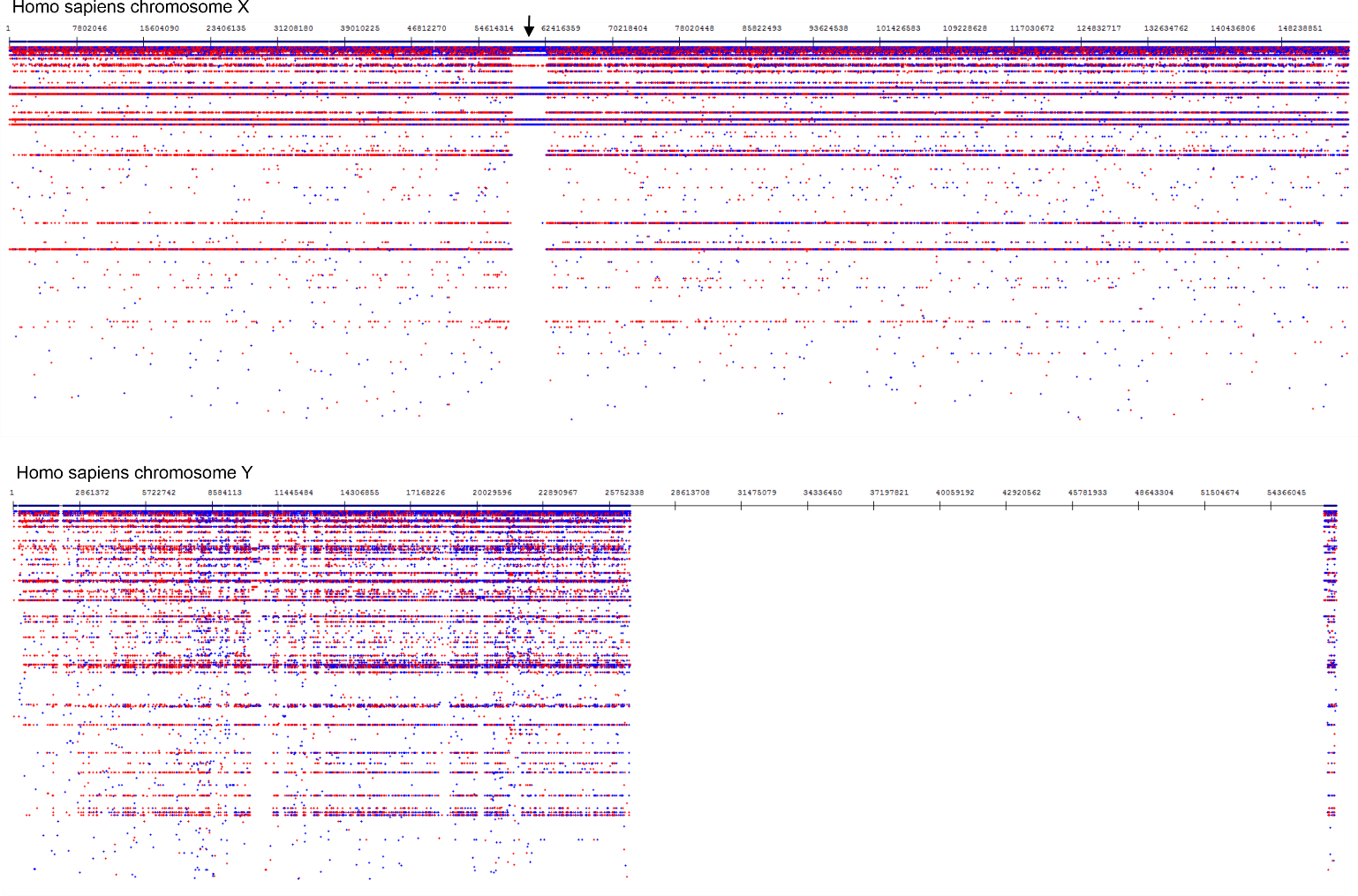


**Figure S4**. Genome-wide profile of all repeats for human chromosome (X and Y) analysis to identify interspersed and clustered repeats. The horizontal axis shows the chromosomal sequence, the vertical axis shows the repeats clustered in a single line. A massive segment of the X chromosome that was not sequenced contains no repeats and was not analyzed. The centromeric region of chromosome X is shown near the points 62416359. The blue and red dots indicate the orientation of the repeat, blue for forward repeats and red for reverse repeats. Analysis parameters (kmer=9; initial length=50; minimum sequence length=550, quicksearch=false). The repeat coverage for chromosome X is 80.06%. For chromosomal Y the repeat coverage is 36.51% for the sequenced part of the chromosome, while the gap in the chromosome is more than 50%.


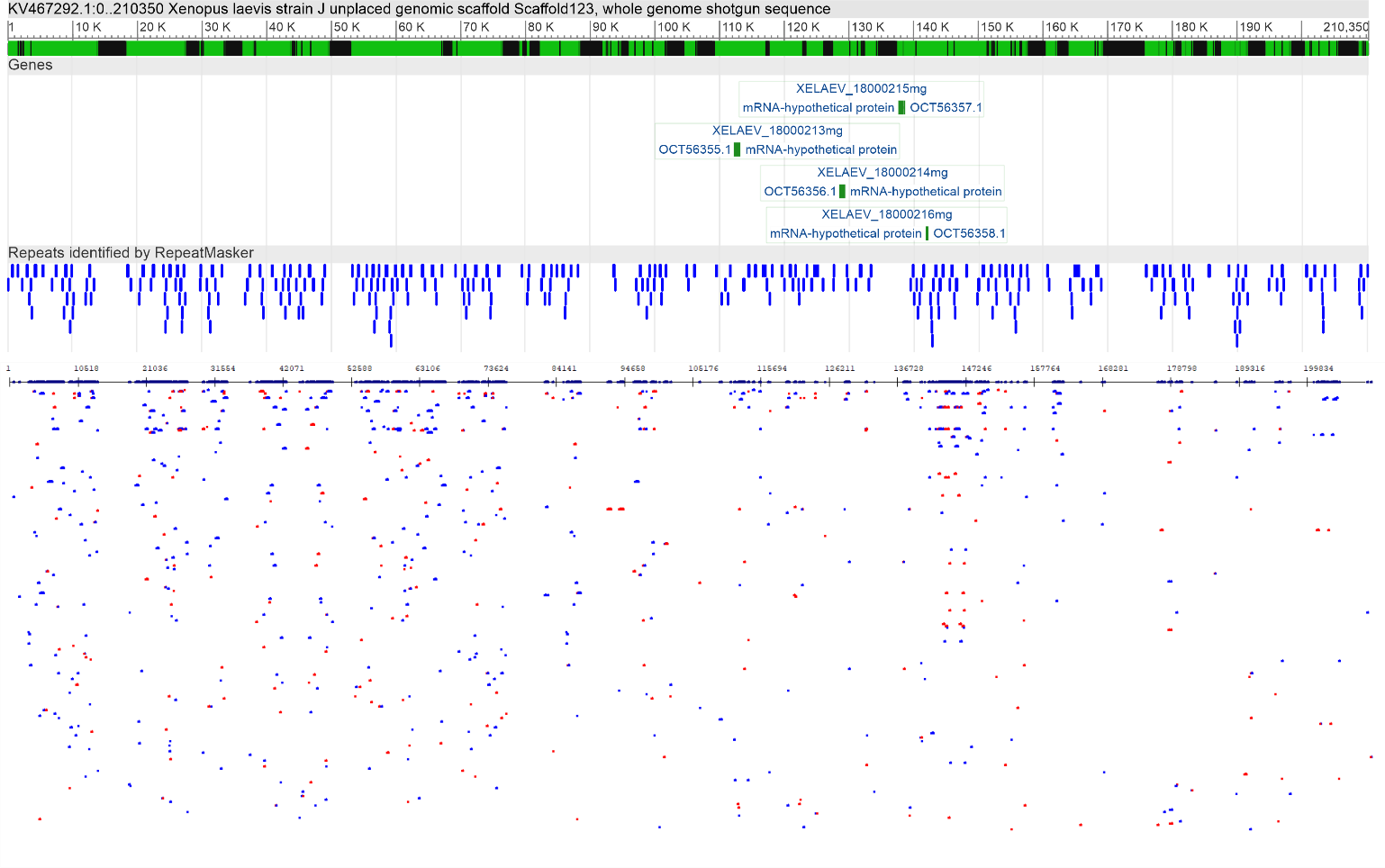


**Figure S5**. Analysis of the genomic scaffold sequence of *Xenopus laevis* (KV467292, <https://www.ncbi.nlm.nih.gov/nuccore/KV467292.1?report=graph>). The upper part of the figure contains a graphical analysis of this site obtained from NCBI GenBank with a graphical view. The lower part of the figure is obtained by a more detailed analysis with clustering of related elements to identify interspersed and clustered repeats.


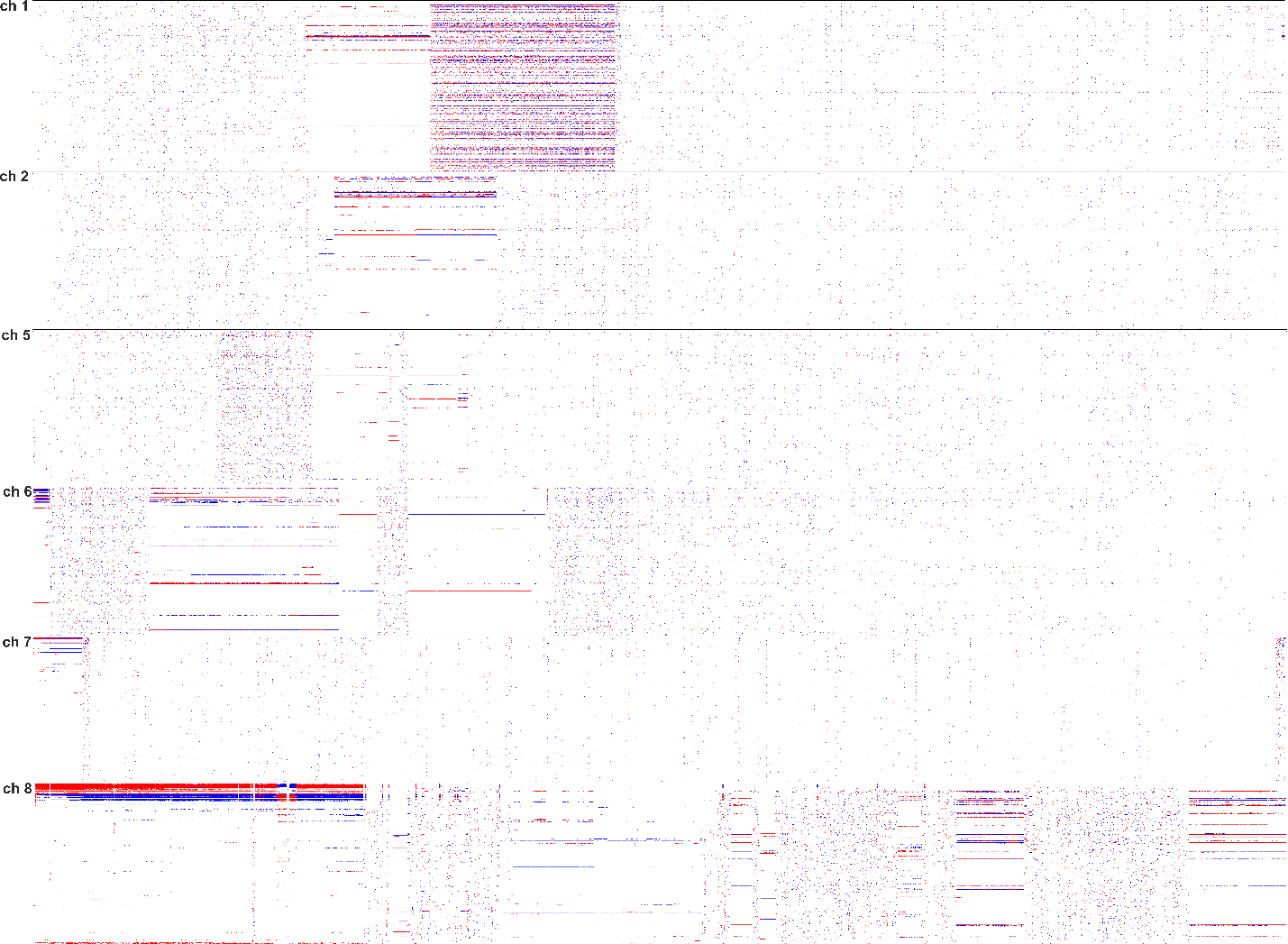


**Figure S6**. Genomic profile of all repeats for the genome of an insect (*Aethina tumida*). The repeat structure for chromosomes 1, 2, 5, 6, 7, and 8 is shown. Parameters to search for repeats, including short *Alu* repeats: kmer=12, Initial string length filter=50, String length filter=100, Quick analysis is false.


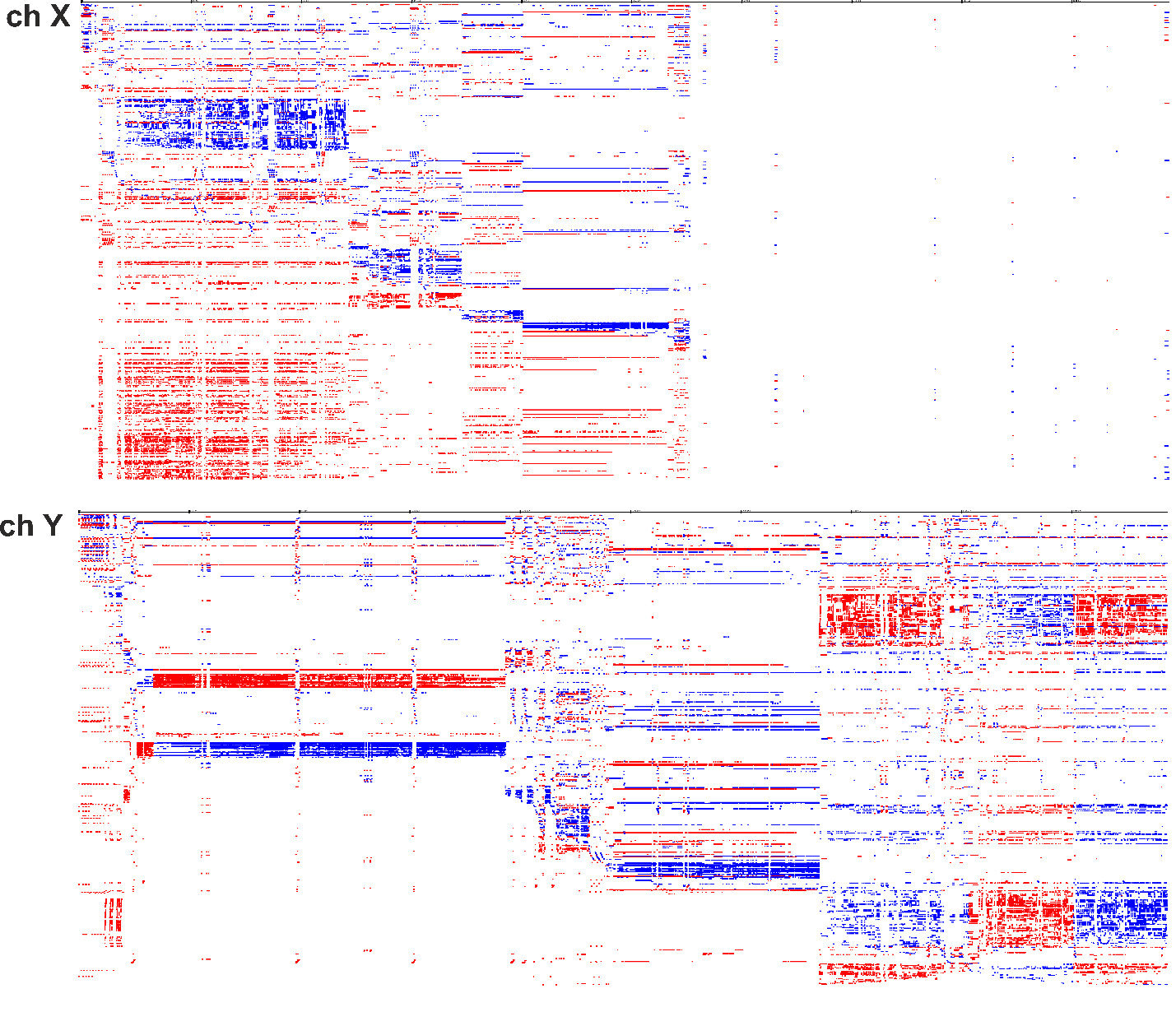


**Figure S7**. Genomic profile of all repeats for the insect (*Coelopa pilipes*) genome. The repeat profile for chromosomes Y and X is shown. Parameters to search for repeats, including short *Alu* repeats: kmer=12, Initial string length filter=50, String length filter=100, Quick analysis is false.


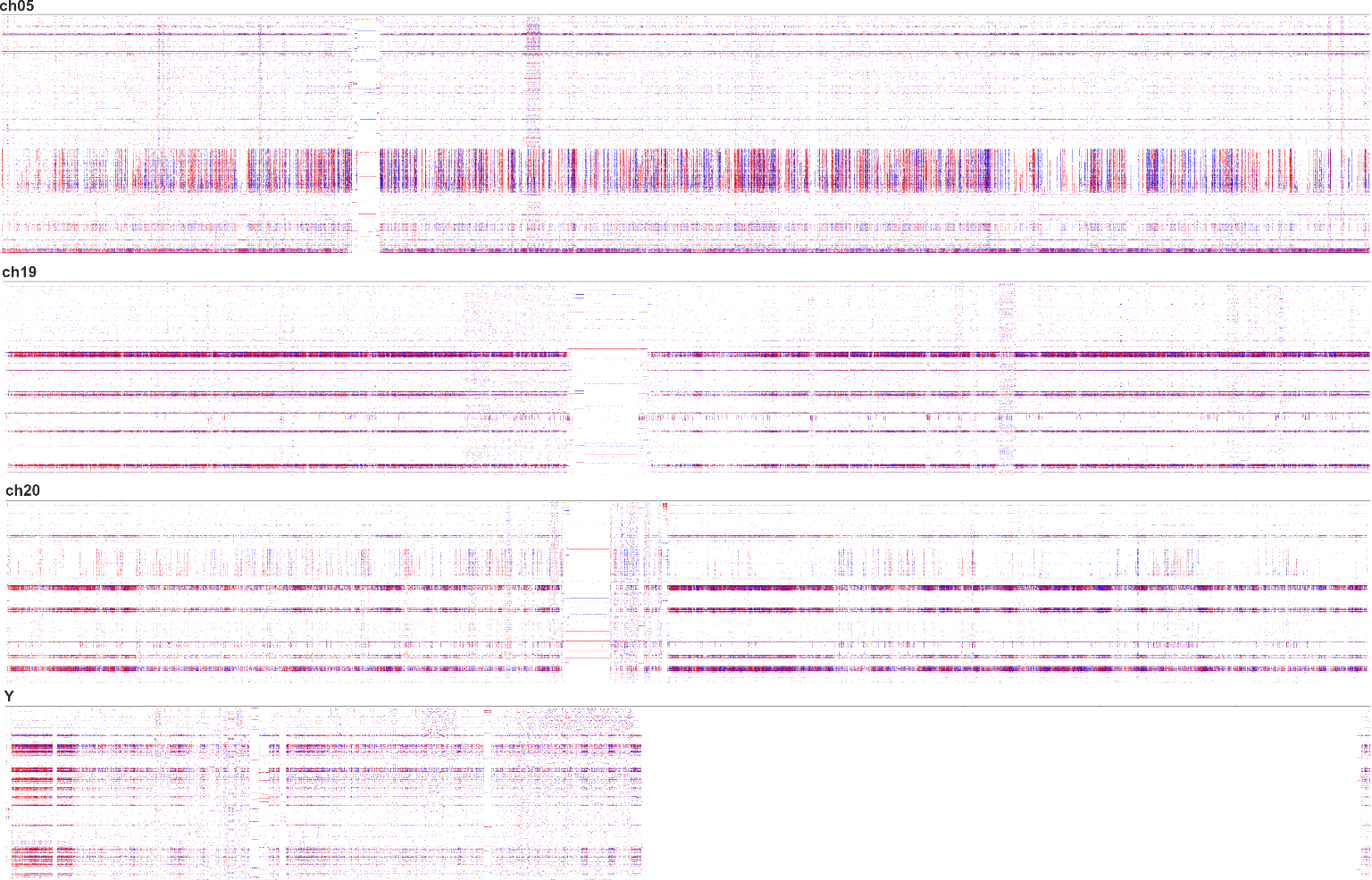


**Figure S8**. Genome-wide profile of all repeats for several human chromosomes (5, 19, 20, and Y) analysis to identify interspersed and clustered repeats. The horizontal axis shows the chromosomal sequence, and the vertical axis shows the repeats clustered in a single line. A massive segment of the X chromosome that was not sequenced contained no repeats and was not analyzed. The blue and red dots indicate the orientation of the repeat, blue for forward repeats, and red for reverse repeats. For chromosomal Y, the repeat coverage is 36.51% for the sequenced part of the chromosome, while the gap in the chromosome is more than 50%.
